# The elephant in the room of ‘planetary health’

**DOI:** 10.1093/eurpub/ckac012

**Published:** 2022-02-24

**Authors:** Johan P Mackenbach

**Affiliations:** Department of Public Health, Erasmus MC, Rotterdam, The Netherlands

As we all know, our planetary environment is deteriorating rapidly. Climate change, biodiversity loss, water pollution, deforestation and other global environmental changes are accelerating and will profoundly affect human health, not only in more vulnerable parts of the world but also in Europe. There is broad consensus that the public health sector needs to urgently engage with these problems,^1^ but there is an elephant in the room that most of us would rather ignore.

These accelerating environmental changes have been caused by a combination of escalating economic growth and escalating population growth, and although increases in consumption per capita have been the most important of the two, increases in human population numbers have also made a significant contribution.^2^ The same will be true in the future: the more the world population grows, the more difficult it will be to reduce the impact of human activity on Earth and to stop further environmental degradation.

The facts speak for themselves. The past increase of the world’s population, from around 1 billion in 1800 to almost 8 billion in 2020, is the result of mortality declines preceding and outpacing fertility declines. Although declining fertility has more recently slowed down population growth, the world’s total population is still growing, and will likely peak at 9 billion, 10 billion or even more during the 21st century, depending on the exact trajectories of mortality and fertility change.

Choosing a low-growth path will have multiple benefits. This will lead to less greenhouse gas emissions, less destruction of other species’ habitats, less pollution, etc. and will also reduce the costs of mitigation and adaptation policies. More fundamentally, while at current population numbers the physical needs of humanity, such as nutrition, can perhaps be met within ‘planetary boundaries’, achievement of more qualitative goals such as high life satisfaction most likely cannot,^3^ so the earlier the world population starts to shrink, the better it is.

Still, population policies are usually not considered as part of climate change strategies, or policies to reduce the speed of biodiversity loss, or other ‘planetary health’ policies. Why is this such an elephant in the room? Misperceptions, such as the incorrect idea that population numbers do not matter much for global environmental change, or the equally incorrect idea that family planning programs are ineffective, may play a role.^4^ But the more important reason probably is that many people feel uncomfortable at the idea of actively promoting a slow-down of population growth, let alone a reduction of human population numbers.

This unease partly stems from the fact that population policies often raise contentious ethical issues around family planning, abortion and immigration, and because one will have to reconcile individual autonomy and the right to procreate with collective interests and the necessity to avoid ecological collapse. Another complicating issue is that population growth currently occurs mainly in countries with low per capita consumption, and that it seems unfair to urge them to limit their reproduction, whereas currently richer countries have never been asked to do the same.^5^

However, this unease must be thought through carefully, particularly by public health professionals, who, after all, are actually increasing the number of people on the Earth. Historically, public health has made a major contribution to reducing mortality, and it is still helping children survive to child-bearing age, or elderly people survive into very old age, which does have an impact on future population numbers and does have downstream consequences for the ecological footprint of humanity. We may try to reassure ourselves by pointing out that mortality decline is usually followed by fertility decline, but is that enough?

I do not think it is. The unfolding ecological crisis requires public health to develop a vision on how it can best contribute to—in the long run, but as soon as possible—reducing the number of people on Earth. Combining public health interventions with family planning programs promoting voluntary birth control is useful, but unlikely to be sufficient, and more radical options should perhaps also be considered.^6^ Use persuasive communication to adjust people’s reproductive preferences? Incentivize people to have less or no children? Slow down the development and deployment of reproductive and life-extending technologies? I do not know, but we should at least look the elephant in the eye.

##  

*Conflicts of interest*: None declared.
